# Effects of chair-based resistance band exercise on physical functioning, sleep quality, and depression of older adults in long-term care facilities: Systematic review and meta-analysis

**DOI:** 10.1016/j.ijnss.2022.12.002

**Published:** 2022-12-26

**Authors:** Ferry Efendi, Santo Imanuel Tonapa, Eka Mishbahatul M. Has, Ken Hok Man Ho

**Affiliations:** a Faculty of Nursing, Universitas Airlangga, Surabaya, Indonesia; b School of Nursing & Midwifery, La Trobe University, Melbourne, Australia; c School of Nursing, Universitas Sam Ratulangi, Manado, Indonesia; d The Nethersole School of Nursing, The Chinese University of Hong Kong, Hong Kong Special Administrative Region, China

**Keywords:** Aged, Exercise movement techniques, Depression, Meta-analysis, Physical functioning, Sleep, Systematic review

## Abstract

**Objective:**

Chair-based resistance band exercise (CRBE) is a simple and safe physical activity for persons with limited mobility. This study aimed to review and analyze CRBE effects on physical functioning, sleep quality, and depression among older adults in long-term care facilities (LTCF).

**Method:**

A systematic search guided by the PRISMA 2020 approach was performed on specific databases: AgeLine, CINAHL, PubMed, Embase, Cochrane Library, Scopus, and Web of Science. The randomized controlled trial studies that trialed CRBE for older adults in LTCF, peer-reviewed articles published in the English language from inception to March 2022 were retrieved. Methodological quality was established using the Physiotherapy Evidence Database scale. The random and fixed effects model were used to generate the pooled effect size.

**Results:**

Nine studies met the eligibility criteria and were synthesized. The results revealed that CRBE significantly promoted the activity of daily living (six studies; *SMD* = 0.30, *P* = 0.001), lung capacity (three studies; *MD* = 40.35, *P* < 0.001), handgrip strength (five studies; *MD* = 2.17, *P* < 0.001), upper limb muscle endurance (five studies; *MD* = 2.23, *P* = 0.012), lower limb muscle endurance (four studies; *MD* = 1.32, *P* < 0.001), upper body flexibility (four studies; *MD* = 3.06, *P* = 0.022), lower body flexibility (four studies; *MD* = 5.34, *P* < 0.001), dynamic balance (three studies; *MD* = −0.35, *P* = 0.011), sleep quality (two studies; *MD* = −1.71, *P* < 0.001), and reduced depression (two studies; *SMD* = −0.33, *P* = 0.035).

**Conclusion:**

The evidence suggests that CRBE improved physical functioning parameters, and sleep quality, and lowers depression among older adults in LTCF. This study could be used to persuade long-term care facilities to allow people with limited mobility to engage in physical activity.

## What is known?

• Physical functioning decline, poor sleep quality, and depression are obstacles that hinder older adults from achieving healthy aging.
• Although research supports that resistance band exercise has a beneficial effect on older adults, most practices are conceptualized for older adults who can walk unaided.

## What is new?

• The present evidence is conclusive that chair-based resistance band exercise (CRBE) positively affects all physical functioning parameters, sleep quality, and depression among older adults in long-term care.
• CRBE in this review is characterized by offering 40–46 min of resistance band exercises on a chair or wheelchair about three times a week, and the program duration spans from 12 weeks to 60 weeks.

## 1 Introduction

The old-age dependence ratio (i.e., the number of individuals aged 65 and over for every 100 persons of working age [ages 20 to 64]) among the Organisation for Economic Co-operation and Development (OECD) countries is anticipated to climb from 28 in 2015 to 58 in 2050 [1], indicating a trend of the global aging population. Aging is well-acknowledged to be a non-modifiable risk factor for long-term care utilization because of chronic diseases or the natural effects of aging on bodily functions [2,3]. It is also reflected in the projection of long-term care (LTC) costs. For example, it was estimated that the LTC cost in China will increase from median values of 39.46, 8.98, and 20.25 billion dollars for mild, moderate, and severe disabilities, respectively, to 141.7, 32.28, and 72.28 billion dollars by 2050 because of the aging population [3]. Although the physical functioning decline has been documented as a predictor of institutionalization in a residential care home [4], a systematic review involving individuals 65 years or older living in residential care homes showed that physical functioning decline among residents could be observed from 3 months to 1.85 years of residence [5]. Rikli and Jones [6] define physical functioning as an individual’s capacity to perform everyday activities safely and independently without undue fatigue. There is unanimous agreement that physical functioning contributes to healthy aging [7]. However, it is crystal clear that the consequences of a decline in physical functioning include poorer quality of life, mental health, and frailty [8].

Exercise or physical activity has the potential for older adults to prevent loss of physical functioning. The WHO’s physical activity guidelines recommend that older adults perform 150–300 min of moderate-intensity physical activity every week to maintain physical functioning [9]. However, older adults in residential care homes did not perform exercise or physical activity sufficiently [8,10]. For example, Australian older adults spent 85% of their time sedentary in a residential care home [8]. Around 64.6% of French older adults in residential care homes did not participate in any exercise programs [10]. Apart from that, over 70% of older adults in long-term care facilities need walking aids for walking or transferring from a bed or chair [11], and around 50%–70% of them use a wheelchair. With these circumstances, many residents in long-term care facilities who rely on walking aids as their primary modality of mobilization are unable to participate in the complex exercise [12].

Physical functioning is commonly demonstrated through the level of independence in performing activities of daily living (ADL) [13]. Apart from the ability to perform ADL independently, critical physical functioning parameters also include lung capacity, muscle endurance, body flexibility, and dynamic balance [6]. The decline in physical functioning will result in multimorbidity and loss of independence, which are also linked to the risk of falls, hospitalization, and mortality [14,15]. Besides the benefit of physical activity, physical inactivity also brought harm, such as decreased sleep quality and increased depression symptoms to older adults. Inadequate exercise or physical activity were risk factors for poor sleep quality [16,17]. There is no definitive definition of sleep quality that is commonly assessed by the Pittsburgh Sleep Quality Index [18]. Meanwhile, it is also well-documented that older adults with poor sleep quality were more likely to have functional limitations [19]. Depression is a common mental disorder worldwide and is popular among older adults with a high dependency on ADL [20]. For older adults, depressive symptoms are linked to an array of negative health outcomes, such as cardiovascular diseases, dementia, and suicide [21,22]. A systematic review with meta-analysis suggested that physical exercise is a feasible intervention to fight depression in older adults [23]. Therefore, promoting physical activity among older adults is essential to achieve healthy aging.

A previous review highlighted that resistance exercise has the largest effect on physical functioning among different types of exercise in older adults [24]. Furthermore, a meta-analysis of randomized controlled trials found performing resistance exercise using an elastic band as an exercise modality significantly improves physical functioning in older adults [25]. Although research supports that resistance exercise has a beneficial effect on older adults, most practices are conceptualized for older adults who can walk unaided [[24], [25], [26]], which may not applicable to older adults in long-term care facilities because of their high prevalence of chair-bounded [11]. Therefore, due to this knowledge-practical gap, there is a need to adapt exercise for older adults with mobility issues in long-term care facilities for promoting their physical functioning. Because of this, many older adults with mobility issues cannot participate in this exercise.

Chair-based resistance band exercise (CRBE) is well regarded as a safe and effective training method for older persons with limited physical ability [27]. CRBE is a modified or elastic band workout performed in a chair-seated position for individuals with limited ability to stand or walk unaided. The resistance level of training can be adjusted flexibly by adjusting the thickness and length of the elastic band. As a sort of muscle-strengthening exercise, this exercise can be utilized to build and maintain muscle strength and power [28]. Several studies have trialed the feasibility and effectiveness of resistance band exercises implemented in a seated position on the chair or as the CRBE for older adults. For example, a study of a 24-week elastic band exercise program for seniors who use a wheelchair showed an improvement in physical functioning markers such as lung capacity, handgrip strength, muscle endurance, body flexibility, and independence in carrying out the activity of daily living [29]. Another trial in Serbia showed that a 12-week chair-based resistance band exercise significantly improves dynamic balance, muscle endurance, and body flexibility [30]. However, some studies also supported opposing findings of the aforementioned trials [31,32], which brings inconsistencies on the effect of CRBE on physical functioning. Apart from those outcomes, CRBE were reported to bring additional health benefits, such as reduced sleep disturbance and depression in older adults with cognitive impairment and dementia [33,34]. Thus, it is necessary to critically review the current evidence of chair-based resistance band exercise on these outcomes. This study aimed to review and analyze the effects of chair-based resistance band exercise on physical functioning, sleep quality, and depression among older adults in long-term care facilities.

## 2 Methods

The review protocol of this study was prospectively registered in the International Prospective Register of Systematic Reviews (PROSPERO): CRD42022320946. This review was reported in accordance with the Preferred Reporting Items for Systematic Reviews and Meta-analysis (PRISMA) guidelines [35].

### 2.1 Search strategy

A systematic search for articles published from 2012 to March 2022 was conducted in AgeLine, CINAHL, PubMed, Embase, Cochrane Library, Scopus, and Web of Science with the help of an experienced medical librarian. The search involved the use of the controlled vocabulary Medical Subject Headings (MeSH) terms “older adults,” “resistance band exercise,” “chair-based,” “physical functioning,” and “randomized controlled trial.” In addition, these keywords were combined with Boolean operators (“AND,” “OR”). Details of the database search strategy deployed in this study are presented Apendices A and B.

The inclusion criteria for articles were determined according to the population, intervention, comparison, outcome, and study design (PICOS) [36]. The inclusion criteria were as follows: 1) population, adults aged ≥65 years in long-term care facilities that refer to a facility that provide a variety of services, both medical and personal care, to people who are unable to live independently, which ranged from the nursing home to continuing care retirement communities [37]; 2)intervention, resistance band exercise performed on the wheelchair or chair and delivered as a single exercise or compound exercise interventions; 3) comparison, the control groups received the usual care or non-exercise interventions; 4) outcome: measured physical functioning, sleep quality, and depression as outcomes with quantifiable scales that had been psychometrically validated; and 5) study design, a randomized controlled trial. The exclusion criteria were: 1) studies that were abstract-only articles, books, theses, conference papers, case reports, protocols, and review articles; 2) studies that were conducted with hospitalized patients; and 3) studies were excluded if they were not published in English and the peer-reviewed journal.

### 2.2 Data evaluation

All retrieved studies were imported into EndNote X9 to exclude duplicate studies. Next, two reviewers (F. Efendi and S. Tonapa) independently screened the remaining studies’ titles and abstracts to assess their eligibility. A third reviewer (E. Has) was invited if there was a difference in opinion between the two reviewers. Finally, the full text was screened and evaluated for eligibility. One reviewer (F. Efendi) extracted data (author name, publication year, country, study design, population/study degree, simulation session, debriefing, simulation modality, interventions and comparisons, outcomes, and tool measurement) from the included studies and discussed it with a second reviewer (S. Tonapa) if further clarification was needed.

The quality of each RCT included in this study was assessed independently by two reviewers (F. Efendi and S. Tonapa) using the Physiotherapy Evidence Database (PEDro) scale [38]. Each of the 11 items on the checklist was used to assess the internal validity and conduct of an RCT. Each trial is scored out of 10, where a score of 9 or more corresponds to excellent quality, a score from 6 to 8 corresponds to good quality, a score from 4 to 5 corresponds to fair quality, and a score less than 4 corresponds to poor quality [39]. If the assessment was not unanimous for each item, the supervisor of the review team was invited to resolve the conflict.

### 2.3 Data analysis

The extracted data from each study were transformed into a pre-calculated effect size with Campbell Collaboration [40], which uses an equation that considers the mean gain scores, pre and post-intervention standard deviation (*SD*), and the correlation coefficient (*r*) between the pre- and post-intervention results. A conservative estimated value (*r* = 0.5) was applied because most studies did not report the *r* values between the pre- and post-intervention scores [40,41].

A mean difference (*MD*) was used to estimate the effect size of each study outcome that has a comparable metric scale. In contrast, standardized mean difference (*SMD*) was used instead for outcomes that do not have a comparable metric scale. An *MD* or *SMD* with a 95% confidence interval (95% CI) calculated the pooled effect size using Comprehensive Meta-Analysis® Version 3.0 (Biostat, Englewood, NJ, USA). The decision to use a random-effects or fixed-effects model was based on the sampling frame between studies pooled together [42]. Additionally, heterogeneity was estimated using Cochran’s *Q*, Tau-squared ($\tau^{2}$), and the *I*-squared (*I*^2^) greater than 75% indicating considerable heterogeneity [43].

A sensitivity analysis was performed using the leave-one study method to ensure the stability of pooled effect size. Finally, we tested the possibility of publication bias via Egger’s regression intercept, and publication bias was identified when *P* < 0.05.

## 3 Results

### 3.1 Identification of studies and study selection

**Fig. 1:**
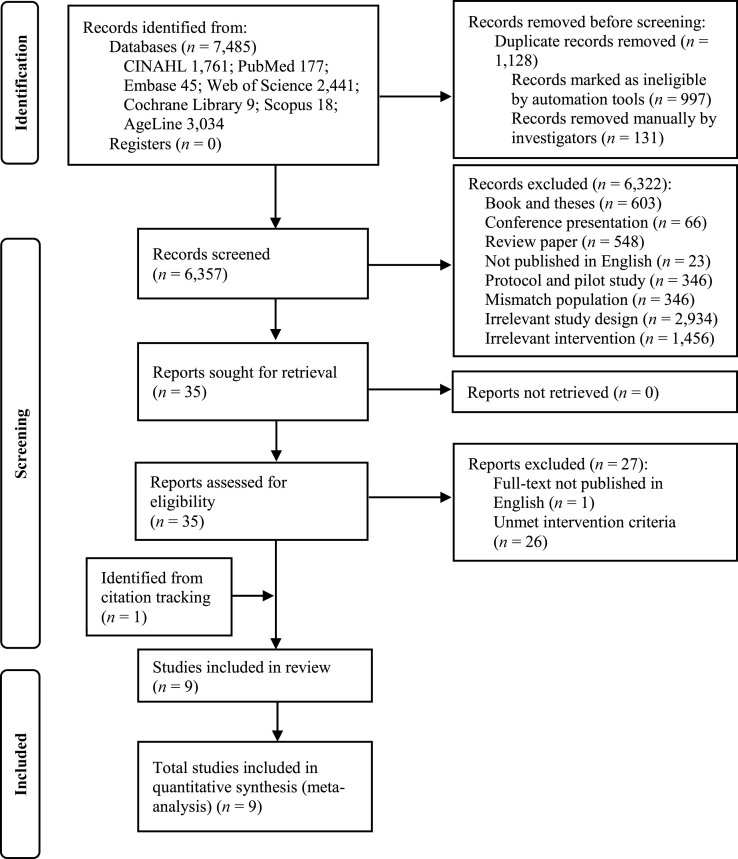
Flowchart of study selection process.

From the seven databases, we initially identified 7,485 articles. Of these, 1,128 were duplicates. The titles and abstracts of the remaining 6,357 studies were screened, and 6,322 were ineligible because they contradict the PICOS criteria. A total of 35 studies were screened in full text to assess eligibility. Of these, 27 studies were excluded: one did not have the full text in English, and 26 did not met the intervention criteria. A total of eight studies met the selection criteria [29,[31], [32], [33], [34],[44], [45], [46]], and one study was found during the full-text screening [30]. Thus, nine studies were included in the meta-analysis (Fig. 1).

### 3.2 The methodological quality of reviewed studies

All studies included in this review were RCTs. Five studies were judged as fair quality (score 4–5), and four demonstrated good quality (score 6–7) (Table 1). All the included RCTs did not perform intention to treat. Only 12.5% of the studies blinded outcome assessors (Appendix C).

**Table 1:** Summary table of evidence for included studies.

Study and location	Mean age, sample size (*n*), and population	Control group	Intervention	Measures, instruments, and time point of measurement	Findings	Quality score
Chen et al., 2015 [29], Taiwan, China, Nursing home	● Both EG + CG: 79.15 ± 7.03, ● EG: (*n =* 59) ● CG: (*n =* 55) ● Using wheelchairs for mobility, living in facility	Usual daily activities	● The program for wheelchair-bound seniors with elastic bands (WSEB) has two levels: the basic and the advanced. The basic WSEB program has three parts (warm-up, aerobic movement, and harmonic stretching), and each part has four elastic band exercises. To make the advanced WSEB program, two fairly difficult exercises were added to each phase of the basic WSEB program. So, the advanced WSEB program is made up of three stages, and each stage has six exercises with an elastic band. Both the basic and the advanced WSEB programs have a 5-minute break right after the aerobic movement phase and take 40 min to finish. The first 12 weeks were spent teaching and practicing the basic level, and the next 12 weeks were spent teaching and practicing the advanced level. ● 24 weeks of three exercise sessions per week (40 min/session)	● Lung capacity/Peak flow meter ● Handgrip strength/Dynamometer ● Upper limbs muscle endurance/Arm curl test ● Lower limbs muscle endurance/Chair-stand test ● Lower body flexibility/Chair sit-and-reach test ● Upper body flexibility/Back scratch test ● Activity of Daily Living (ADL)/Barthel index ● Baseline - 24 weeks	● All outcome variables were significant improvement in the EG and the EG was greater than CG. ● Only upper body flexibility was insignificant improvement between EG and CG.	7
Stojanović et al., 2021 [30], Serbia, Geriatric center	● EG: 75.7 ± 8.9, (*n =* 86) ● CG: 74.5 ± 8.2, (*n =* 82) ● Female older adults	Usual daily activities	● Elastic band resistance training consists of 10-min of warm-up; 40-min of elastic band exercise; 5–10 min of cool-down. ● 12 weeks of twice exercise sessions per week (60 min/session). RPE level was 4–5, which could be reduced by decreasing the elastic band grip breadth or changing the elastic band color.	● Handgrip strength/Dynamometer ● Upper limbs muscle endurance/Arm curl test ● Lower limbs muscle endurance/Chair-stand test ● Upper body flexibility/Back scratch test ● Lower body flexibility/Chair sit-and-reach test ● Dynamic balance/Timed Up and Go test (TUG) ● Baseline - 12 weeks	All of outcome variables were significant improvement in the EG.	5
Furtado et al., 2020 [31], Portugal, Center of health and social support institution	● EG: 81.00 ± 4.79, (*n =* 20) ● CG: 80.93 ± 10.01, (*n =* 59) ● Frail or pre-frail older women	No exercise	● Chair elastic-band muscle strength exercises consist of three phases: 5-min of warm-up; muscle- strengthening exercises using elastic band; cool-down. ● Performed exercises 2–3 times a week (45 min/session) for 28 weeks. Intensity of elastic band increasing from level one (yellow band) at weeks 1–14 to level 2–3 at weeks 15–28. The intensity of RPE was kept at 4–6 points, and the HRmax was 45%–50%.	● ADL/Katz index ● Baseline - 14 weeks–28 weeks	ADL was insignificant improvement in the EG and did not improve between the EG and CG.	4
Rieping et al., 2019 [32], Portugal, Social and health care support center	● EG: 83.47 ± 4.92, (*n =*15) ● CG: 80.85 ± 10.86, (*n =*13) ● Female older adults aged >75	No exercise, but encouraged to engage in complementary activities	● Chair elastic-band muscle strength exercises consist of three phases: 5-min of warm-up; muscle- strengthening exercises using elastic band; cool-down. ● 14 weeks of three exercise sessions per week (45 min/session). Exercise intensity was maintained at 10 submaximal repetitions with an RPE of 6–8.	● Upper limbs muscle endurance/Arm curl test ● Lower limbs muscle endurance/Chair-stand test ● Dynamic balance/TUG ● ADL/Katz index ● Baseline - 14 weeks	The EG was insignificant improvement of upper body strength, dynamic balance, and ADL.	4
Chen et al., 2016 [33], Taiwan, China, Nursing home	● EG: 80.79 ± 0.91, (*n =*73) ● CG: 81.37 ± 0.84, (*n =* 65) ● Wheelchair-bound older adults with cognitive impairment	Ordinary daily activities	● WSEB consists of three phases: warm-up; aerobic motion; harmonic strength. Each session has four elastic band exercise and 5-min break after aerobic motion phase. The advanced WSEB program adds two tough exercises to each phase of the standard WSEB program, so six elastic band exercise in each phase. ● Performed exercises three times a week (40 min/session) for 24 weeks.	● Lung capacity/Peak flow meter ● Handgrip strength/Dynamometer ● Upper limbs muscle endurance/Arm curl test ● Upper body flexibility/Back scratch test ● Lower body flexibility/Chair sit-and-reach test ● ADL/Barthel Index ● Baseline – 24 weeks	All of outcome variables were significant improvement in the EG.	6
Chen et al., 2017 [34], Taiwan, China, Nursing home	● EG: 80.70 ± 8.00, (*n =* 65) ● CG: 81.60 ± 6.70, (*n =* 62) ● Older adults with dementia, using wheelchair for mobility	Usual daily activities	● The resistance band exercises were conducted in the following two sequences: volunteer-led sessions for the first six months (Stage I) followed by DVD-guided sessions for the next nine months (Stage II). The basic program of resistance exercise consists of three phases: warm-up; aerobic motion; harmonic strength. Each session has four elastic band exercise and 5-min break after aerobic motion phase. The advanced program adds two tough exercises to each phase of the standard WSEB program, thus six elastic band exercise in each phase. ● 60 weeks of three weekly exercise sessions (40 min/session)	● Depression/the Cornell Scale for Depression in Dementia (CSDD) ● Baseline - 60 weeks	The EG was significantly less depressed than the CG.	6
Cancela et al., 2017 [44] Spain, Daycare	● EG: 84.92 ± 3.40 (*n=*12) ● CG: 89.00 ± 5.43 (*n=*12) ● Very old people (aged >80)	Joints mobility routine	● Resistance bands exercise in both upper and lower limbs, including flexion-extension, abduction-adduction position. ● Performed exercises three times a week (45 min/session) for 12 weeks. Each week, the amount of repetitions each set was raised without a rest in between.	● Handgrip strength/Dynamometer ● Dynamic balance/TUG ● ADL/Barthel index ● Baseline - 13 weeks	The EG had improvement of ADL, handgrip strength, and dynamic balance.	5
Chen et al., 2015 [45], Taiwan, China, Nursing home	● Both EG + CG: 79.15 ± 7.03 ● EG: (*n =* 59) ● CG: (*n =* 55) ● Wheelchair-bound older adult, living in facility	Regular daily activities	● WSEB consists of three phases: warm-up; aerobic motion; harmonic strength. Each phase has four elastic band exercise and 5-min break after aerobic motion phase. The advanced WSEB program adds two tough exercises to each phase of the standard WSEB program, so six elastic band exercise in each phase. ● Performed exercises three times a week (40 min/session) for 24 weeks.	● Sleep quality/the Pittsburgh Sleep Quality Index (PSQI) ● Depression/the Taiwanese Depression Questionnaire (TDQ) ● Baseline - 24 weeks	The EG had longer sleep durations, better habitual sleep efficiencies, and less depression than the CG.	5
Chen et al., 2016 [46], Taiwan, China, Nursing home	● EG: 78.83 ± 7.69, (*n =* 64) ● CG: 80.10 ± 6.35, (*n =* 63) ● Using wheelchairs for mobility, living in facility	Usual activities	● The basic WSEB consists of three phases with four exercises in each phase: warm-up (wrist rotation, chest expansion, knee elevation, and shuttlecock bouncing), aerobic motion (boxing, hand raising, trunk waving, and stepping and pushing), and harmonic stretching (directing traffic, pulling forward, attacking the flank, and separating the thighs). Two relatively difficult exercises were added in each phase of the basic program to form the advanced program (such as kicking the stone, pulling arms, spreading the wings, whipping out a sword, lifting legs, and reaching the calf). The resistance band exercises were conducted in the following two sequences: volunteer-led sessions for the first 24 weeks (Stage I) followed by DVD-guided sessions for the next 24 weeks (Stage II). ● 48 weeks of three weekly exercise sessions (40 min/session)	● Lung capacity/Peak flow meter ● Handgrip strength/Dynamometer ● Upper limbs muscle endurance/Arm curl test ● Lower limbs muscle endurance/Chair-stand test ● ADL/Barthel index ● Sleep quality/the Pittsburgh Sleep Quality Index (PSQI) ● Baseline - 24 weeks–48 weeks	All of outcome variables were significant improvement in the EG and the EG was greater than CG.	6

*Note:* EG = experimental group. CG = control group. NR = not reported. RPE = rating perceived exertion.

### 3.3 Study characteristics

These studies were published between 2015 and 2021. The smallest sample size was 23 [44], and the largest sample size was 168 [30]. A total of 878 older adults were included in the nine studies. Of them, 453 were in an intervention group, while 425 were in a control group. The mean ages of the participants ranged from 75.7 years to 84.9 years in the intervention group, whereas the control group ranged from 74.5 years to 89 years.

Older adults with various conditions were involved in the included studies, including healthy, frailty, cognitive impairment, dementia, and wheelchair user. There is no report about adverse events related to the intervention in all included studies. Included studies were carried out in various types of long-term care facilities such as nursing homes (*n* = 6), daycare (*n* = 1), geriatric centers (*n* = 1), and centers of health and social support institutions (*n* = 2). Among the nine RCTs, six trials provided a usual or routine daily activity for participants in the control group, two studies did not administer any exercise, and one study provided a joint mobility routine. In the present study, most studies’ protocols for CRBE comprised three sessions: warm-up, muscle-strengthening using a resistance band, and cool-down. The average time of each session based on the study outcome was around 40–46 min. The CRBE is performed about three times per week on average. The average program duration for chair-based resistance exercise is about 24 weeks. Moreover, some of the studies reported that exercise intensity was adjusted at some point by increasing exercise repetition or tension level of the resistance band. Detailed characteristics of the included studies are shown in Table 1.

### 3.4 Effects of CRBE on physical functioning

#### 3.4.1 Activity daily living

Six studies examined the effects of CRBE on independence in activity daily living measured by a Barthel index [29,33,44,46] and Katz index [31,32]. Under the random-effects model, the forest plot demonstrated the pooled *SMD* was 0.30 (95% CI: 0.11, 0.48, *P* = 0.001) (Appendix D), with low heterogeneity ($\tau^{2}$ = 0.01, *Q* = 6.70, *df* = 6, *I*^2^ = 10.4%) (Table 2). These results suggest that chair-based resistance band exercise statistically significantly improves independence in activity daily living compared with routine care.

**Table 2:** Overall effects of chair-based resistance bands exercise.

Outcomes	*n*	Pooled effect size			Heterogeneity				Egger test
		*SMD/MD*	*Z*	*P*	*Q*-statistic	*P*	*I*^2^ (%)	Tau^2^	*P*
Physical Functioning									
Activity daily living	6	0.30	3.18	0.001	6.70	0.350	10.4	0.01	0.385
Lung capacity	3	40.35	5.54	<0.001	1.27	0.737	0	0	0.633
Handgrip strength	5	2.17	4.49	<0.001	2.22	0.817	0	0	0.997
Upper limb muscle endurance	5	2.23	2.51	0.012	26.19	<0.001	80.9	3.70	0.502
Lower limb muscle endurance	4	1.32	4.08	<0.001	0.65	0.957	0	0	0.662
Upper body flexibility	4	3.06	2.29	0.022	1.62	0.140	0	0	0.209
Lower body flexibility	4	5.34	3.99	<0.001	3.61	0.140	42.1	3.61	0.032
Dynamic balance	3	−0.35	−2.54	0.011	1.06	0.590	0	0	0.389
Sleep Quality	2	−1.71	−3.93	<0.001	0.39	0.822	0	0	0.929
Depression	2	−0.33	−2.11	0.035	4.49	0.106	55.4	0.04	0.121

*Note*: *n*, numbers of studies analyzed. *SMD* = standard mean difference. *MD* = mean difference.

#### 3.4.2 Lung capacity

Three studies examined the effects of chair-based resistance band exercise on lung capacity, measured by a peak flow meter [29,33,46]. Under the fixed-effects model, the forest plot demonstrated the pooled *MD* was 40.35 (95% CI: 26.07, 54.63, *P* < 0.001) (Appendix E), with low heterogeneity ($\tau^{2}$ = 0.00, *Q* = 1.27, *df* = 3, *I*^2^ = 0.0%) (Table 2). These results suggest that CRBE statistically significant improved lung capacity compared with routine care.

#### 3.4.3 Handgrip strength

Five studies examined the effects of CRBE on handgrip strength, measured by a dynamometer [29,30,33,44,46]. Under the random-effects model, the forest plot demonstrated the pooled *MD* was 2.17 (95% CI: 1.22, 3.11, *P* < 0.001) (Appendix E), with low heterogeneity ($\tau^{2}$ = 0.00, *Q* = 2.22, *df* = 5, *I*^2^ = 0.0%) (Table 2). These results suggest that CRBE statistically significant improved handgrip strength compared with routine care.

#### 3.4.4 Upper limb muscle endurance

Five studies examined the effects of CRBE on upper limb muscle endurance, measured with an arm curl test [29,30,32,33,46]. Under the random-effects model, the forest plot demonstrated the pooled *MD* was 2.23 (95% CI: 0.49, 3.97, *P* = 0.012) (Appendix E), with considerable heterogeneity ($\tau^{2}$ = 3.70, *Q* = 26.19, *df* = 5, *I*^2^ = 80.9%) (Table 2). These results suggest that CRBE statistically significant improved upper limb muscle endurance compared with routine care.

#### 3.4.5 Lower limb muscle endurance

Four studies examined the effects of CRBE on lower limb muscle endurance, measured with a chair-stand test [29,30,32,46]. Under the random-effects model, the forest plot demonstrated the pooled *MD* was 1.32 (95% CI: 0.69, 1.96, *P* < 0.001) (Appendix E), with low heterogeneity ($\tau^{2}$ = 0.00, *Q* = 0.65, *df* = 4, *I*^2^ = 0.0%) (Table 2). These results suggest that CRBE statistically significant improved lower limb muscle endurance more than routine care.

#### 3.4.6 Upper body flexibility

Four studies examined the effects of CRBE on upper body flexibility measured with a back scratch test [29,32,33,46]. Under the random-effects model, the forest plot demonstrated the pooled *MD* was 3.06 (95% CI: 0.44, 5.68, *P* = 0.022) (Appendix E), with low heterogeneity ($\tau^{2}$ = 0.00, *Q* = 1.62, *df* = 4, *I*^2^ = 0.0%) (Table 2). These results suggest that CRBE statistically significant improves upper body flexibility compared with routine care.

#### 3.4.7 Lower body flexibility

Four studies examined the effects of CRBE on lower body flexibility measured with a chair sit-and-reach test [29,30,33,46]. Under the random-effects model, the forest plot demonstrated the pooled MD was 5.34 (95% CI: 2.71, 7.96, *P* < 0.001) (Appendix E), with moderate heterogeneity ($\tau^{2}$ = 3.61, *Q* = 6.92, *df* = 4, *I*^2^ = 42.1%) (Table 2). These results suggest that CRBE statistically significant improves lower body flexibility compared with routine care.

#### 3.4.8 Dynamic balance

Three studies examined the effects of CRBE on dynamic balance with a Timed Up and Go test (TUG) [32,44] and 8-Foot Up-and-Go test [30]. Under the random-effects model, the forest plot demonstrated the pooled *MD* was −0.35 (95% CI: −0.61, −0.08, *P* = 0.011) (Appendix E), with low heterogeneity ($\tau^{2}$ = 0.00, *Q* = 1.06, *df* = 2, *I*^2^ = 0.0%) (Table 2). These results suggest that CRBE statistically significant improves dynamic balance compared with routine care.

### 3.5 Effects of CRBE on sleep quality

Two studies examined the effects of CRBE on sleep quality measured by the Pittsburgh Sleep Quality Index [45,46]. Under the fixed-effects model, the forest plot demonstrated the pooled *MD* was −1.71 (95% CI: −2.56, −0.86, *P* < 0.001) (Appendix D), with low heterogeneity ($\tau^{2}$ = 0.00, *Q* = 0.39, *df* = 2, *I*^2^ = 0.0%) (Table 2). These results suggest that CRBE significantly statistically significant improved sleep quality compared with routine care.

### 3.6 Effects of CRBE on depression

Two studies examined the effects of CRBE on depression measured by the Taiwanese Depression Scale (TDS) [45] and the Cornell Scale for Depression in Dementia (CSDD) [34]. Under the random-effects model, the forest plot demonstrated the pooled *SMD* was −0.33 (95% CI: −0.64, −0.02, *P* = 0.035) (Appendix D), with moderate heterogeneity ($\tau^{2}$ = 0.04, *Q* = 4.49, *df* = 2, *I*^2^ = 55.4%) (Table 2). These results suggest that CRBE statistically significant reduced depression compared with routine care.

### 3.7 Publication bias

The results of the Egger regression test for publication bias analysis are provided in Table 2. Finding regarding the effects of CRBE on lower body flexibility should be interpreted with caution because there was evidence of publication bias (*P* = 0.032). However, there was no indication of publication bias for the other outcomes.

### 3.8 Sensitivity analysis

The sensitivity analyses were carried out using the leave one study method (Appendix F,G). After removing studies with the heaviest weight, it was indicated that no one study alone was likely to substantially skew or drive the *MD* or *SMD* in either direction.

## 4 Discussion

This systematic review and meta-analysis scrutinized evidence on the effects of CRBE. The pooled effect size demonstrated that CRBE positively affects all physical functioning parameters, sleep quality, and depression among older adults in long-term care. In addition, in the included RCTs, the age category of older adults between middle-old and oldest-old has various degrees of health conditions (frailty, cognitive impairment, dementia, wheelchair users, and healthy). All nine RCTs offered 40–46 min of resistance band exercises in a chair or wheelchair performed about three times a week, with a program duration spanning 12 weeks–60 weeks. The dosage of exercise in this current review corresponds to WHO physical activity recommendations, which advise older adults to engage in muscle-strengthening at least two days a week [9,47]. In sum, the CRBE is suitable and feasible and can be another alternative exercise that can help older adults with various health conditions promote their physical and psychological health.

This review revealed that CRBE improves physical functioning parameters comprised of lung capacity, handgrip strength, limb muscle endurance, body flexibility, and dynamic balance, which align with previous trials [30,33,44,46]. First, an increased lung capacity is possible because the CRBE featured abdominal breathing and movements similar to exercises for lung expansion (chest expansion, trunk waving, and arms drawing) [33]. The increase in lung capacity was noteworthy because of its essential role in making sure older adults can execute the activity of daily living without exhaustion [48]. Next, handgrip strength was improved because resistance band exercise procedures consist of contracting motions such as pulling and holding resistance bands that may build strength in the muscles as the user pulls against the bands [44,46]. Regarding enhanced muscle endurance, the exercise positions also consisted of pushing and pulling the elastic band with several intensities at the upper and lower limbs. The intensity involved the number of set repetitions of the elastic band exercise and the resistance level of the elastic band. The CRBE also enhances muscle flexibility because this exercise incorporates movements such as flexion, extension, and adduction of body joints in the upper or lower body, which may decrease musculotendinous and musculoarticular stiffness [30,49]. Moreover, the increased dynamic balance after CRBE may be linked to the increase in lower body muscle endurance and lower body flexibility [50].

Performing regular CRBE with sufficient dosage may preserve physical functioning, which is necessary for older adults independently in executing an activity of daily living. Some activities warrant older adults to be physically fit. For instance, walking and mobilization require good dynamic balance. A greater lung capacity is needed for older adults to perform their activities longer without fatigue. Also, good body flexibility and muscle strength enable them to dress independently, get up from the toilet, and climb stairs. The improvement of older adults’ activities of daily living after this exercise echoed the previous studies [29,[31], [32], [33],^44^,^46^].

Older adults who were involved in CRBE experienced an improvement in sleep quality. This finding is noteworthy because older adults often have sedentary lifestyles, such as repetitive daily routines and a lack of physical activity, which may be associated with sleep disturbances [51]. Poor sleep habits, such as excessive time spent in bed and sleeping during the day, exacerbated the problem of sleep disturbances, particularly for this specific group of older adults with mobility issues [52]. Sleep quality has improved since the CRBE programs stimulate older adults to be physically active, which reduces the time to sleep during day time. However, it should be noted that the observed improvement in sleep quality across the studies was seen after 6–12 months which indicated that sleep quality could improve if older adults performed exercise regularly within a long-term period [45,46].

The present review found that the effects of CRBE are not limited to physical health but also reduce depression in older adults. The reduction of depressive symptoms after the exercise program is probably due to multiple mechanisms that started from increasing older adults’ physical functioning and reducing their dependence levels. Next, the increase of independence in activities of daily living brings additional effects on older adults that build their self-esteem and sense of control which may help reduce depressive symptoms [53,54]. This finding brings an implication to the broader global community because the WHO estimated that more than 264 million people worldwide suffer from depression, with older adults accounting for around 7% of those affected. Thus, it is recommended that older adults who cannot participate in complex exercises perform CRBE to reduce depressive symptoms.

## 5 Limitations

This current systematic review and meta-analysis have limitations to consider. In this review, only studies published in English were included, which might have excluded important data from papers published in other languages. Some of the findings in each outcome listed below should be regarded with caution. First, in accordance with the Cochrane guidelines, the current study cannot perform subgroup analysis and meta-regression to explore the source of heterogeneity in the result on upper limb muscle endurance, owing to the relatively small sample size in this review (≤4 studies) [55]. Due to the small sample size, the stability of sleep quality and depression findings cannot be ruled out through sensitivity analysis which should be interpreted cautiously. Last, findings regarding lower body flexibility should be interpreted carefully because publication bias occurred.

## 6 Conclusion

Physical functioning decline, poor sleep quality, and depression are obstacles that hinder older adults with impaired mobility from achieving healthy aging. The cumulative evidence is conclusive that CRBE positively affects all physical functioning parameters, the activity of daily living, sleep quality, and depression among older adults in long-term care. With the evidence from the present study, CRBE may serve as evidence for LTCF to empower residents with impaired mobility to perform physical activity. But more research in the future is needed to figure out how CRBE affect older adults with different levels of disability.

## Funding

This study was funded by 10.13039/501100008463Universitas Airlangga, Indonesia through Article Review scheme number 200/UN3.15/PT/2022.

## Data availability

The authors declare the absence of shared data in the present study.

## CRediT authorship contribution statement

**Ferry Efendi:** Conceptualization, Methodology, Data Curation, Investigation, Writing- original draft, Writing-review & editing, Supervision, Funding acquisition. **Santo Imanuel Tonapa:** Conceptualization, Methodology, Data curation, Investigation, Formal analysis, Writing-original draft, Writing-review & editing. **Eka Mishbahatul M. Has:** Conceptualization, Methodology, Writing-original draft, Writing-review & editing. **Ken Hok Man Ho:** Conceptualization, Methodology, Writing-original draft, Writing-review & editing.

## Declaration of competing interest

No conflicts of interest to declare.
