# Supplementary material for: Effects of chair-based resistance band exercise on physical functioning, sleep quality, and depression of older adults in long-term care facilities: Systematic review and meta-analysis
Source: Int J Nurs Sci. 2022 Dec 26;10(1):72–81. doi: 10.1016/j.ijnss.2022.12.002 (PMC9969069; doi:10.1016/j.ijnss.2022.12.002)
Supplement: Multimedia component 1 [file mmc1.docx]

座椅辅助阻力带运动对老年人身体功能、睡眠质量和抑郁的影响：系统评价

Ferry Efendi, Santo Imanuel Tonapa, Eka Mishbahatul M.Has, Ken Hok Man Ho

【摘要】

**目的** 座椅辅助阻力带运动(Chair-based resistance band exercise，CRBE)是一种简单安全的运动，适合行动不便的人。本研究系统评价CRBE对长期护理机构(LTCF)老年人身体功能、睡眠质量和抑郁的影响。

**方法**  根据PRISMA（2020）指南和综合评价方法，对AgeLine, CINAHL, PubMed, Embase, Cochrane Library, Scopus和 Web of Science等数据库进行检索。检索时间为建库至2022年3月，纳入对慢性疾病老年人进行CRBE的随机对照试验研究，且经同行评审的英文文献。采用物理治疗证据数据库量表（the Physiotherapy Evidence Database, PEDro)对纳入文献进行质量评价，随机效应模型和固定效应模型用于生成集合效应量。

**结果**  共9篇文章符合纳入标准。结果显示，CRBE可促进日常生活活动(6项研究；*SMD*=0.30, *P*=0.001)，肺活量(3项研究；*MD*=40.35, *P**＜*0.001)，握力(5项研究；*MD*=2.17, *P＜*0.001)，上肢肌肉耐力(5项研究；*MD*=2.23, *P=*0.012)，下肢肌肉耐力(4项研究；*MD*=1.32, *P＜*0.001)，上肢柔韧性(4项研究；*MD*=3.06, *P=*0.022)，下体柔韧性(4项研究；*MD*=5.34, *P＜*0.001)，动态平衡(3项研究；*MD*=−0.35, *P*=0.011)、睡眠质量(2项研究；*MD*=−1.71, *P＜*0.001)，并减轻抑郁状况 (2项研究；*SMD*=−0.33, *P*=0.035)。

**结论** 现有证据表明CRBE改善了长期慢性疾病老年人的身体功能参数、睡眠质量，并减轻抑郁状况。这项研究可用以敦促长期护理机构让行动不便的老年人参与体育活动。

【**关键词**】老年人；身体锻炼；抑郁；Meta分析；身体功能；睡眠；系统评价

**通信作者：**Ferry Efendi，E-mail:[ferry-e@fkp.unair.ac.id](mailto:ferry-e@fkp.unair.ac.id)
