# Supplementary material for: Effects of chair-based resistance band exercise on physical functioning, sleep quality, and depression of older adults in long-term care facilities: Systematic review and meta-analysis
Source: Int J Nurs Sci. 2022 Dec 26;10(1):72–81. doi: 10.1016/j.ijnss.2022.12.002 (PMC9969069; doi:10.1016/j.ijnss.2022.12.002)
Supplement: Multimedia component 2 [file mmc2.docx]

Appendix A PICO worksheet

| Items | Object | Keywords |
| --- | --- | --- |
| Population | Older adults | Aged, aging, elder*, older, older adult*, old, older person*, older population, older people, old-age, senior*, residence characteristics, communit* |
| Intervention | Chair-based Resistance Bands Exercise | Chair*, chair based*, seat*, sit*, sat, resistance bands exercise, resistance bands training, elastic band exercise, resistance cords exercise |
| Comparison | Treatment as usual | N/A |
| Outcome | Physical fitness and health outcomes | physical fitness, muscle strength, body strength, muscle endurance, body flexibility, lung capacity, aerobic endurance, cardiovascular function, pulmonary function, physical activit*, activity daily living, ADL, sleep quality, sleep, depressive disorder, depression |
| Study design | Experimental study | Research, randomized controlled trial, randomized controlled study, randomly assigned |
