# Supplementary material for: Effects of chair-based resistance band exercise on physical functioning, sleep quality, and depression of older adults in long-term care facilities: Systematic review and meta-analysis
Source: Int J Nurs Sci. 2022 Dec 26;10(1):72–81. doi: 10.1016/j.ijnss.2022.12.002 (PMC9969069; doi:10.1016/j.ijnss.2022.12.002)
Supplement: Multimedia component 3 [file mmc3.docx]

Appendix B. Database search terms

| Databases | Set | Search terms | Items found |
| --- | --- | --- | --- |
| CINAHL | 1 | TI ( aged OR aging OR elder* OR older OR olders OR older adult* OR old OR older person* OR older population OR older people OR old adult* OR old people OR old person* OR old population OR old-age OR old-aged OR senior* AND Residence Characteristics OR communit* ) OR AB ( aged OR aging OR elder* OR older OR olders OR older adult* OR old OR older person* OR older population OR older people OR old adult* OR old people OR old person* OR old population OR old-age OR old-aged OR senior* AND Residence Characteristics OR communit* ) | 810,764 |
|  | 2 | TI ( Chair* OR chair based* OR seat* OR sit* OR sat AND Resistance Bands Exercise OR Resistance Bands Training OR Elastic Band Exercise OR Resistance Cords Exercise ) OR AB ( Chair* OR chair based* OR seat* OR sit* OR sat AND Resistance Bands Exercise OR Resistance Bands Training OR Elastic Band Exercise OR Resistance Cords Exercise ) | 294,486 |
|  | 3 | TI ( physical fitness OR muscle strength OR body strength OR muscle endurance OR body flexibility OR lung capacity OR aerobic endurance OR cardiovascular function OR pulmonary function OR physical activit* OR activity daily living OR ADL AND sleep quality OR sleep AND depressive disorder OR depression ) OR AB ( physical fitness OR muscle strength OR body strength OR muscle endurance OR body flexibility OR lung capacity OR aerobic endurance OR cardiovascular function OR pulmonary function OR physical activit* OR activity of daily living OR ADL AND sleep quality OR sleep AND depressive disorder OR depression ) | 304,424 |
|  | 4 | TI ( Research OR Randomized controlled trial OR randomized controlled study OR randomly assigned ) OR AB (Randomized controlled trial OR randomized controlled study OR randomly assigned ) | 806,128 |
|  | 5 | 1 AND 2 AND 3 AND 4 | 1,761 |
| PUBMED | 1 | (("aged"[MeSH Terms] OR (("aged"[Title/Abstract] OR "aging"[Title/Abstract] OR "ageing"[Title/Abstract] OR "elder"[Title/Abstract] OR "elders"[Title/Abstract] OR "elderly"[Title/Abstract] OR "older"[Title/Abstract] OR "olders"[Title/Abstract] OR "older adult"[Title/Abstract] OR "older adults"[Title/Abstract] OR "old"[Title/Abstract] OR "older person"[Title/Abstract] OR "older persons"[Title/Abstract] OR "older population"[Title/Abstract] OR "older people"[Title/Abstract] OR "old adult"[Title/Abstract] OR "old adults"[Title/Abstract] OR "old people"[Title/Abstract] OR "old person"[Title/Abstract] OR "old persons"[Title/Abstract] OR "old population"[Title/Abstract] OR "old age"[Title/Abstract] OR "old aged"[Title/Abstract] OR "senior"[Title/Abstract] OR "seniors"[Title/Abstract]) AND "aged"[MeSH Terms])) AND ("Residence Characteristics"[MeSH Terms] OR "communit*"[Title/Abstract])) | 129,909 |
|  | 2 | chair*[Title/Abstract] OR chair based*[Title/Abstract] OR seat*[Title/Abstract] OR sit*[Title/Abstract] OR sat[Title/Abstract] AND exercis*[Title/Abstract] OR activit*[Title/Abstract] OR train*[Title/Abstract] OR program*[Title/Abstract] OR resistance band* exercise[Title/Abstract] OR resistance bands training[Title/Abstract] OR elastic bands exercise[Title/Abstract] OR elastic bands training[Title/Abstract] OR (exercise*[MeSH Terms]) | 4,844,070 |
|  | 3 | (("physical fitness"[MeSH Terms] OR ("physical fitness"[Title/Abstract] OR "muscle strength"[Title/Abstract] OR "body strength"[Title/Abstract] OR "muscle endurance"[Title/Abstract] OR "body flexibility"[Title/Abstract] OR "lung capacity"[Title/Abstract] OR "aerobic endurance"[Title/Abstract] OR "cardiovascular function"[Title/Abstract] OR "pulmonary function"[Title/Abstract] OR "physical activit*"[Title/Abstract] OR "activity of daily living"[Title/Abstract] OR "ADL"[Title/Abstract])) AND (("sleep quality"[Title/Abstract] OR "sleep"[MeSH Terms] AND "depressive disorder"[MeSH Terms] OR "depression"[Title/Abstract]) | 387,225 |
|  | 4 | ((research[Title/Abstract] ) OR (Random Allocation[MeSH Terms])) OR (randomized controlled trial*[Title/Abstract] OR randomized controlled study[Title/Abstract] OR randomly assigned[Title/Abstract]) | 2,157,738 |
|  | 5 | 1 AND 2 AND 3 AND 4 | 177 |
| Embase | 1 | (aged:ab,ti OR aging:ab,ti OR elder*:ab,ti OR older:ab,ti OR olders:ab,ti OR 'older adult*':ab,ti OR old:ab,ti OR 'older person*':ab,ti OR 'older population':ab,ti OR 'older people':ab,ti OR 'old adult*':ab,ti OR 'old people':ab,ti OR 'old person*':ab,ti OR 'old population':ab,ti OR 'old age':ab,ti OR 'old aged':ab,ti OR senior*:ab,ti) AND 'residence characteristics':ab,ti OR communit*:ab,ti | 814,050 |
|  | 2 | (chair*:ab,ti OR 'chair based*':ab,ti OR seat*:ab,ti OR sit*:ab,ti OR sat:ab,ti) AND 'resistance bands exercise':ab,ti OR 'resistance bands training':ab,ti OR 'elastic band exercise':ab,ti OR 'resistance cords exercise':ab,ti | 45 |
|  | 3 | (('physical fitness':ab,ti OR 'muscle strength':ab,ti OR 'body strength':ab,ti OR 'muscle endurance':ab,ti OR 'body flexibility':ab,ti OR 'lung capacity':ab,ti OR 'aerobic endurance':ab,ti OR 'cardiovascular function':ab,ti OR 'pulmonary function':ab,ti OR 'physical activit*':ab,ti OR 'activity daily living':ab,ti OR adl:ab,ti) AND 'sleep quality':ab,ti OR sleep:ab,ti) AND 'depressive disorder':ab,ti OR depression:ab,ti | 521,971 |
|  | 4 | research:ab,ti OR 'randomized controlled trial':ab,ti OR 'randomized controlled study':ab,ti OR 'randomly assigned':ab,ti | 2,554,724 |
|  | 5 | 1 AND 2 AND 3 AND 4 | 45 |
| Web of Science | 1 | ((TI=(older adult* or elder* or old* people or old* person* or old* population or senior*)) OR AB=((older adult* or elder* or old* people or old* person* or old* population or senior*))) OR KP=(aged or elderly or older adult*) | 959,474 |
|  | 2 | (((TI=(chair* OR 'chair based OR seat* OR sit* OR sat AND 'resistance bands exercise' OR 'resistance bands training' OR 'elastic bands exercise' OR 'elastic bands training')) OR AB=(chair* OR 'chair based OR seat* OR sit* OR sat AND 'resistance bands exercise' OR 'resistance bands training' OR 'elastic bands exercise' OR 'elastic bands training')) OR KP=(elastic bands exercise* or bands exercise*)) AND AB=(chair* OR 'chair based OR seat* OR sit* OR sat AND 'resistance bands exercise' OR 'resistance bands training' OR 'elastic bands exercise' OR 'elastic bands training') | 2,951,656 |
|  | 3 | (TI=(physical fitness OR muscle strength OR body strength OR muscle endurance OR body flexibility OR lung capacity OR aerobic endurance OR cardiovascular function OR pulmonary function OR physical activit* OR activity daily living OR ADL AND sleep quality OR sleep AND depressive disorder OR depression)) OR AB=(physical fitness OR muscle strength OR body strength OR muscle endurance OR body flexibility OR lung capacity OR aerobic endurance OR cardiovascular function OR pulmonary function OR physical activit* OR activity daily living OR ADL AND sleep quality OR sleep AND depressive disorder OR depression) | 858,259 |
|  | 4 | ((TI=(research or randomized controlled trial* or intervention study)) OR AB=(research or randomized controlled trial* or intervention study)) OR KP=(randomization or RCT*) | 3,516,251 |
|  | 5 | 1 AND 2 AND 3 AND 4 | 2,441 |
| Cochrane Library (Trials) | 1 | (Aged or aging* or ageing* or elder* or old* or senior* or old-age or old-aged):ti,ab,kw | 219,477 |
|  | 2 | (chair* or chair based* or seat* or sit* or sat and resistance bands exercise* or resistance bands training or resistance band program* or elastic bands exercise* or elastic bands training or elastic bands program*):ti,ab,kw | 131,313 |
|  | 3 | (physical fitness or muscle strength or muscle endurance or body strength or body flexibility or lung capacity or aerobic endurance or cardiovascular function or pulmonary function or physical activit* or activity of daily living or ADL):ti,ab,kw and MeSH descriptor: [Sleep] explode all trees or (sleep quality or sleep):ti,ab,kw and MeSH descriptor: [Depressive Disorder] explode all trees or (depression):ti,ab,kw | 1,500 |
|  | 4 | MeSH descriptor: [Random Allocation] explode all trees or (research or randomized controlled trial* or intervention study):ti,ab,kw | 954,745 |
|  | 5 | 1 AND 2 AND 3 AND 4 | 9 |
| Scopus | 1 | ( TITLE-ABS-KEY ( aged OR aging OR elder* OR older OR olders ) OR TITLE-ABS-KEY ( older AND adult* OR old ) OR TITLE-ABS-KEY ( older AND person* ) OR TITLE-ABS-KEY ( older AND population ) OR TITLE-ABS-KEY ( older AND people ) OR TITLE-ABS-KEY ( old AND adult* ) OR TITLE-ABS-KEY ( old AND people ) OR TITLE-ABS-KEY ( old AND person* ) OR TITLE-ABS-KEY ( old AND population ) OR TITLE-ABS-KEY ( old-age OR old-aged OR senior* ) AND TITLE-ABS-KEY ( residence AND characteristics ) OR TITLE-ABS-KEY ( communit* ) ) | 327,230 |
|  | 2 | ( TITLE-ABS-KEY ( chair* OR chair-based* OR seat* OR sit* OR sat ) AND TITLE-ABS-KEY ( resistance AND bands AND exercise ) OR TITLE-ABS-KEY ( resistance AND bands AND training ) OR TITLE-ABS-KEY ( elastic AND band AND exercise ) OR TITLE-ABS-KEY ( resistance AND cords AND exercise ) ) | 3,182 |
|  | 5 | 1 AND 2 | 18 |
| AgeLine | 1 | TI ( aged OR aging OR elder* OR older OR olders OR older adult* OR old OR older person* OR older population OR older people OR old adult* OR old people OR old person* OR old population OR old-age OR old-aged OR senior* AND Residence Characteristics OR communit* ) OR AB ( aged OR aging OR elder* OR older OR olders OR older adult* OR old OR older person* OR older population OR older people OR old adult* OR old people OR old person* OR old population OR old-age OR old-aged OR senior* AND Residence Characteristics OR communit* ) | 138,790 |
|  | 2 | TI ( Chair* OR chair based* OR seat* OR sit* OR sat AND Resistance Bands Exercise OR Resistance Bands Training OR Elastic Band Exercise OR Resistance Cords Exercise ) OR AB ( Chair* OR chair based* OR seat* OR sit* OR sat AND Resistance Bands Exercise OR Resistance Bands Training OR Elastic Band Exercise OR Resistance Cords Exercise ) | 63,524 |
|  | 3 | TI ( physical fitness OR muscle strength OR body strength OR muscle endurance OR body flexibility OR lung capacity OR aerobic endurance OR cardiovascular function OR pulmonary function OR physical activit* OR activity daily living OR ADL AND sleep quality OR sleep AND depressive disorder OR depression ) OR AB ( physical fitness OR muscle strength OR body strength OR muscle endurance OR body flexibility OR lung capacity OR aerobic endurance OR cardiovascular function OR pulmonary function OR physical activit* OR activity of daily living OR ADL AND sleep quality OR sleep AND depressive disorder OR depression ) | 28,427 |
|  | 4 | TI ( Research OR Randomized controlled trial OR randomized controlled study OR randomly assigned ) OR AB (Randomized controlled trial OR randomized controlled study OR randomly assigned ) | 34,568 |
|  | 5 | 1 AND 2 AND 3 AND 4 | 3,034 |
| Last database search: March 24th 2022 | | | |
