# Supplementary material for: Effects of chair-based resistance band exercise on physical functioning, sleep quality, and depression of older adults in long-term care facilities: Systematic review and meta-analysis
Source: Int J Nurs Sci. 2022 Dec 26;10(1):72–81. doi: 10.1016/j.ijnss.2022.12.002 (PMC9969069; doi:10.1016/j.ijnss.2022.12.002)
Supplement: Multimedia component 4 [file mmc4.docx]

Appendix C. Quality appraisal

| Study | Cancela et al. (2017) | Chen, Li, et al. (2015) | Chen, Huang, et al. (2015) | K. M. Chen et al. (2016) | Furtado et al. (2020) | Rieping et al. (2019) | M. C. Chen et al. (2016) | Chen et al. (2017) | Stojanović et al. (2021) | Summary (%) |
| --- | --- | --- | --- | --- | --- | --- | --- | --- | --- | --- |
| *Items appraisal* |  |  |  |  |  |  |  |  |  |  |
| 1. Eligibility criteria were specified | 1 | 1 | 1 | 1 | 1 | 1 | 1 | 1 | 1 | 100% |
| 2. Subjects were randomly allocated to groups (in a crossover study, subjects were randomly allocated an order in which treatments were received) | 1 | 1 | 1 | 1 | 1 | 0 | 1 | 1 | 1 | 87.5% |
| 3. Allocation was concealed | 1 | 1 | 0 | 1 | 0 | 0 | 1 | 1 | 1 | 62.5% |
| 4. The groups were similar at baseline regarding the most important prognostic indicators | 0 | 1 | 1 | 1 | 1 | 1 | 1 | 1 | 1 | 87.5% |
| 5. There was blinding of all subjects | 0 | 0 | 0 | 0 | 0 | 0 | 0 | 0 | 0 | 0% |
| 6. There was blinding of all therapists who administered the therapy | 0 | 0 | 0 | 0 | 0 | 0 | 0 | 0 | 0 | 0% |
| 7. There was blinding of all assessors who measured at least one key outcome | 0 | 1 | 0 | 0 | 0 | 0 | 0 | 0 | 0 | 12.5% |
| 8. Measures of at least one key outcome were obtained from more than 85% of the subjects initially allocated to groups | 1 | 1 | 1 | 1 | 0 | 1 | 1 | 1 | 1 | 87.5% |
| 9. All subjects for whom outcome measures were available received the treatment or control condition as allocated or, where this was not the case, data for at least one key outcome was analyzed by “intention to treat” | 0 | 0 | 0 | 0 | 0 | 0 | 0 | 0 | 0 | 0% |
| 10. The results of between-group statistical comparisons are reported for at least one key outcome | 1 | 1 | 1 | 1 | 1 | 1 | 1 | 1 | 1 | 100% |
| 11. The study provides both point measures and measures of variability for at least one key outcome | 1 | 1 | 1 | 1 | 1 | 1 | 1 | 1 | 0 | 87.5% |
| *Total* | 5 | 7 | 5 | 6 | 4 | 4 | 6 | 6 | 5 |  |

*Note:* Total PEDro scores of 0-3 are considered ‘poor’, 4-5 ‘fair’, 6-8 ‘good’, and 9-10 ‘excellent’, it is important to note that these classifications have not been validated.
