# Supplementary figures and images for: Effects of chair-based resistance band exercise on physical functioning, sleep quality, and depression of older adults in long-term care facilities: Systematic review and meta-analysis
Source: Int J Nurs Sci. 2022 Dec 26;10(1):72–81. doi: 10.1016/j.ijnss.2022.12.002 (PMC9969069; doi:10.1016/j.ijnss.2022.12.002)

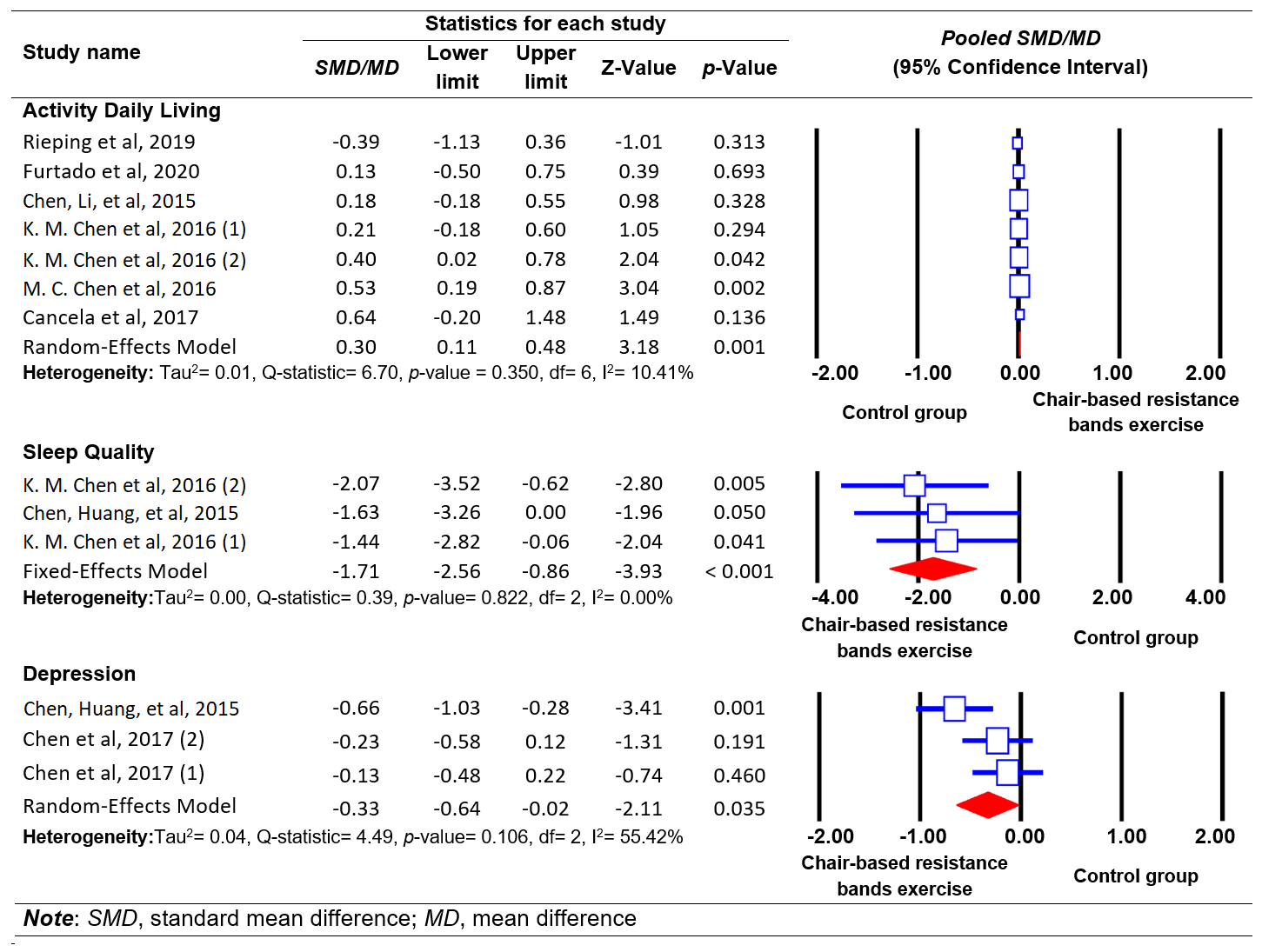


Appendix D Forest plot for ADL, Sleep quality, and Depression

Supplement: Multimedia component 5 [file mmc5.docx]

Appendix E. Forest plot for Physical Functioning
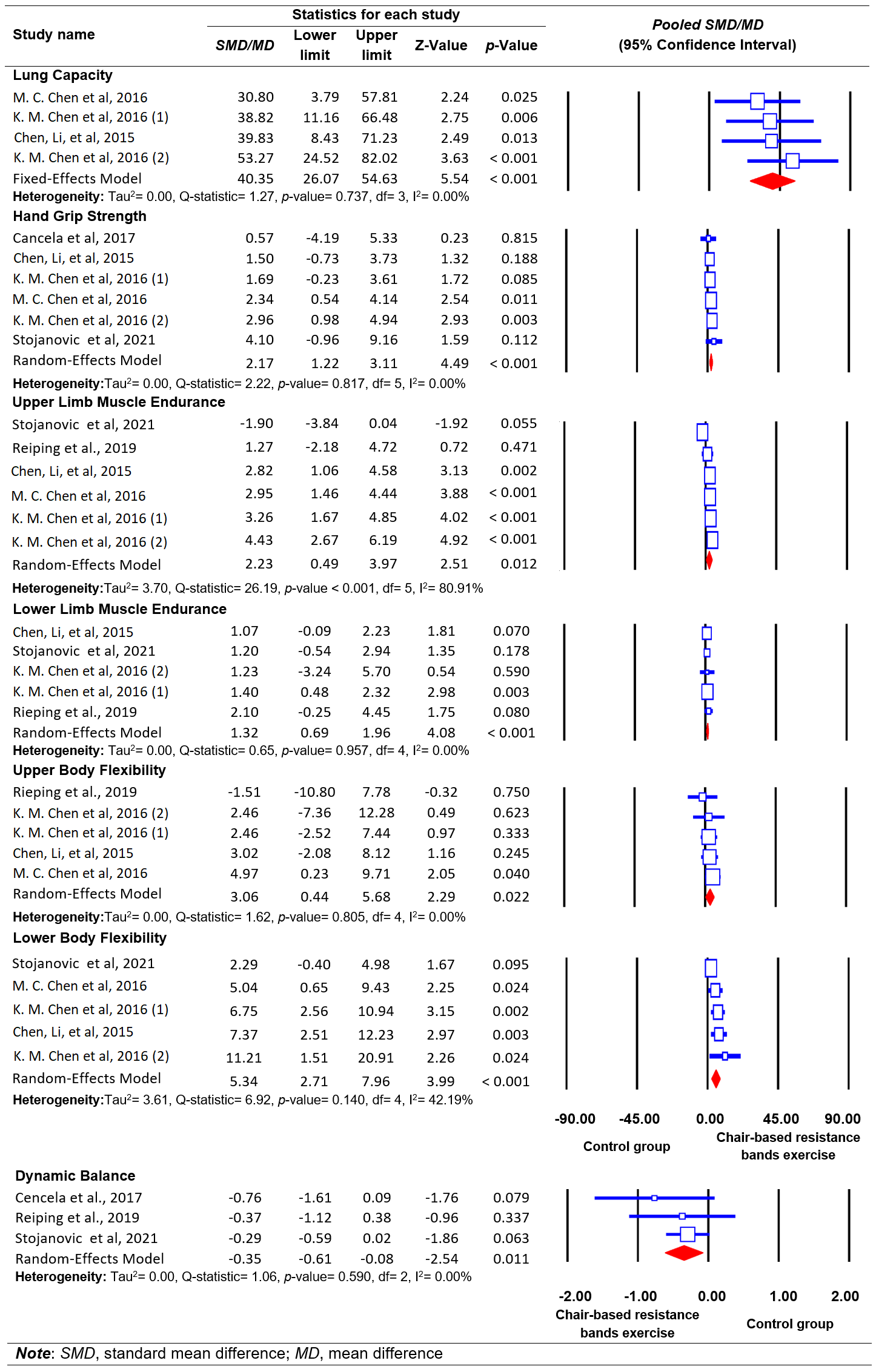

Supplement: Multimedia component 6 [file mmc6.docx]

Appendix F. Sensitivity Analysis for Physical Functioning
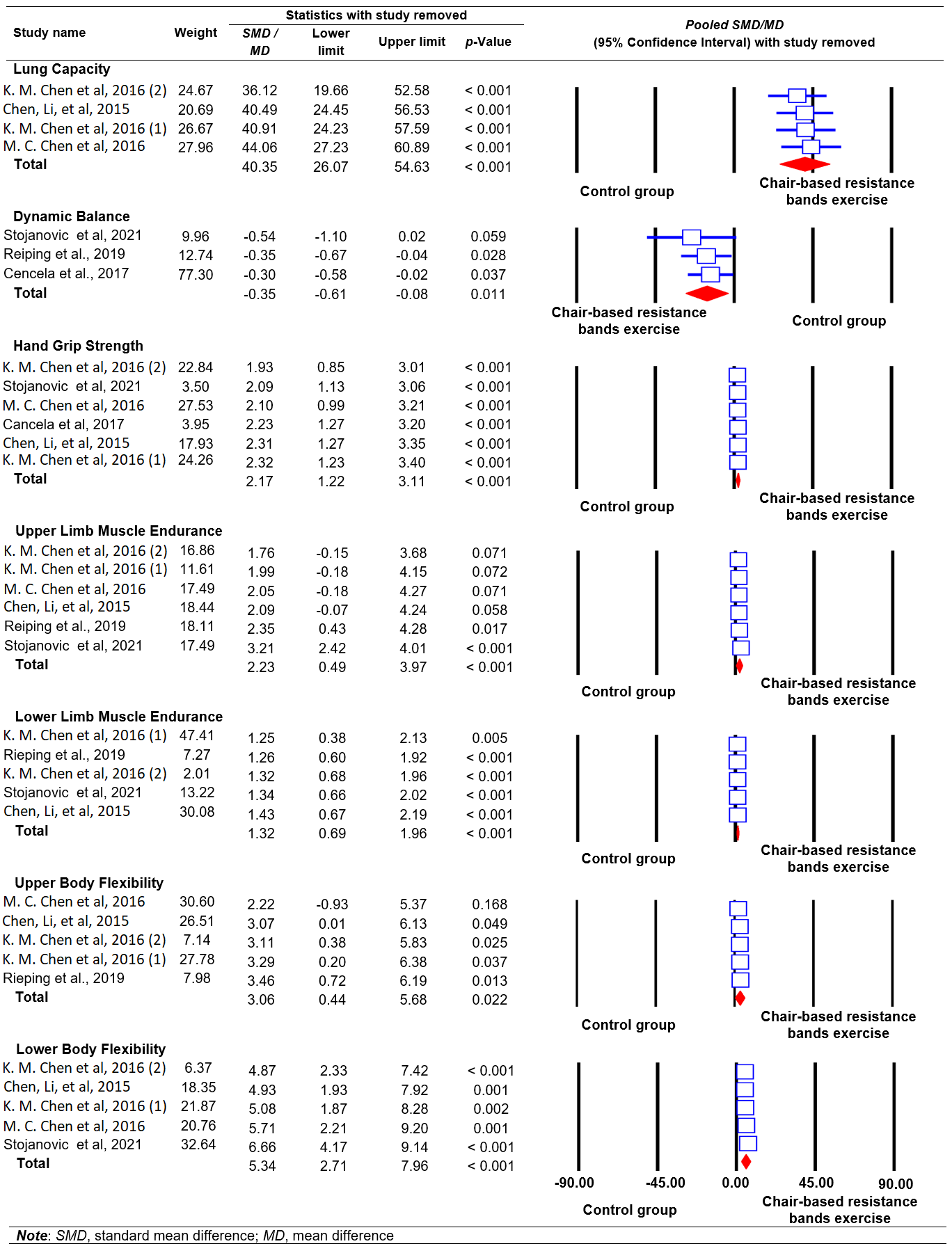

Supplement: Multimedia component 7 [file mmc7.docx]

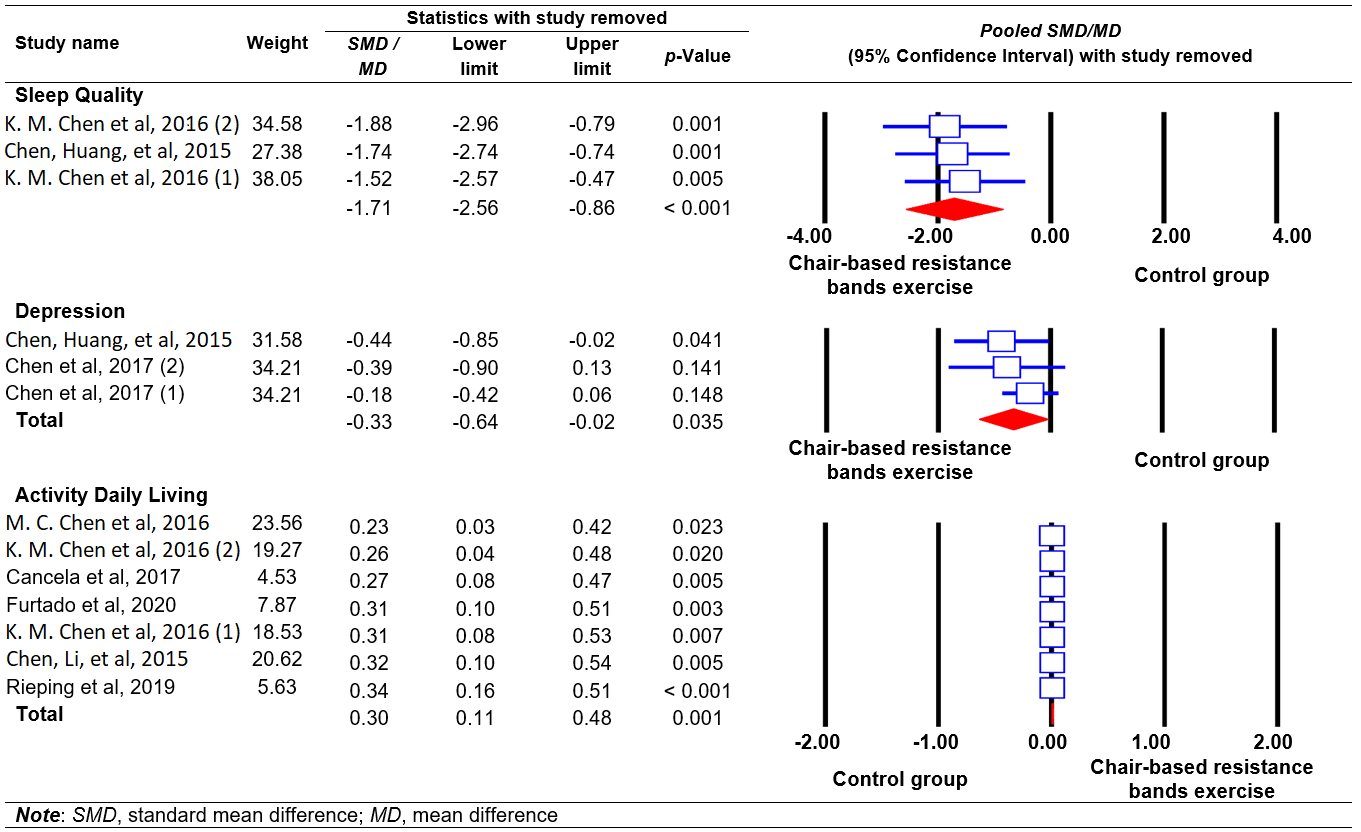


Appendix G. Sensitivity Analysis for Sleep Quality, Depression, and Activity of Daily Living

Supplement: Multimedia component 8 [file mmc8.docx]
